# Pharmacotherapies for smoking cessation among Indigenous Peoples: A systematic review of randomized controlled trials

**DOI:** 10.1016/j.pmedr.2026.103460

**Published:** 2026-03-30

**Authors:** Salma Mahmoodianfard, Emma Lynn Bates, Javad Heshmati, Maalik Sheraly, Jake Miles, Sarah Visintini, Richard San Cartier, Hassan Mir

**Affiliations:** a University of Ottawa Heart Institute, Ottawa, Ontario, Canada; b Faculty of Medicine, University of Ottawa, Ottawa, Ontario, Canada; c Faculty of Health Sciences, University of Ottawa, Ottawa, Ontario, Canada; d Berkman Library, University of Ottawa Heart Institute, Ottawa, Ontario, Canada; e Maamwesying Indigenous Health Care Services, Cutler, Ontario, Canada; f Ottawa Model for Smoking Cessation, Ottawa, Ontario, Canada

**Keywords:** Indigenous population, Smoking cessation, Pharmacotherapy, Culturally tailored interventions, Systematic review

## Abstract

Indigenous Peoples experience disproportionately high rates of commercial tobacco use and smoking-related illness. Although pharmacotherapies are effective in the general population, their effectiveness among Indigenous communities remains uncertain. This systematic review synthesized randomized controlled trials (RCTs) evaluating pharmacotherapies for smoking cessation among Indigenous adults.

**Methods:** MEDLINE, Embase, CENTRAL, APA PsycINFO, Informit Indigenous Collection, Bibliography of Indigenous Peoples in North America, and Global Health were searched in June–September 2024, with an update of all but Informit on May 28, 2025. Eligible studies were RCTs assessing pharmacotherapies, alone or combined with behavioral or culturally tailored supports for smoking cessation among Indigenous adults. Due to heterogeneity, findings were synthesized narratively.

**Results:** Five RCTs (*n* = 1633 participants) from American Indian, Alaska Native, and Māori populations incorporating nicotine replacement therapy, bupropion, varenicline, and cytisine met inclusion criteria. At six months, self-reported 7-day point-prevalence abstinence ranged from 12.0% to 41.4% and biochemically verified abstinence from 6.9% to 22.6%. Adherence was generally high, and adverse events were mild.

**Conclusions:** Limited but suggestive evidence indicates that pharmacotherapies, particularly when paired with supportive or culturally tailored interventions, may aid smoking cessation among Indigenous adults. Interpretation is constrained by heterogeneity and high attrition. Further community-led, culturally grounded RCTs are needed.

## Highlights

• Pharmacotherapies provide modest support for smoking cessation in Indigenous adults.
• Trials reported high medication adherence and mostly mild adverse events.
• Evidence is limited, with clear gaps in Indigenous-focused cessation research.

## 1 Introduction

Tobacco smoking is a leading global cause of preventable illness and mortality, accounting for 8.7 million deaths and about 230 million disability-adjusted life years in 2019 (He et al., 2021). The implementation of strong anti-tobacco policies from the World Health Organization (WHO) has resulted in global declines in tobacco use (World Health Organization, 2003); however; prevalence remains high in certain populations. Indigenous Peoples worldwide experience disproportionately high rates of tobacco use and they carry a substantially greater burden of smoking-related illness and mortality compared with the general population (Singh et al., 2024). In the United States; American Indian/Alaska Native (AI/AN) adults smoke at more than twice the national rate (27.1% vs 12.5%) (Cornelius et al., 2022). In Canada; smoking prevalence among Indigenous Peoples is approximately two to five times higher than among non-Indigenous Canadians (e.g.; 53.5% among First Nations; 70–84% in Nunavut) (Health Canada, 2023; First Nations Information Governance Centre, 2018; Government of Nunavut, Department of Health, 2024). Similar patterns are evident in Australia (41% vs ∼14% among non-Indigenous adults) (Australian Institute of Health and Welfare; National Indigenous Australians Agency, 2020) and New Zealand (17.1% vs 6.8% nationally) (Primary Health Care Centre, 2024). These consistently elevated rates across countries and regions underscore persistent inequities in tobacco use and the limited impact of conventional tobacco control measures for Indigenous Peoples.

The persistence of high smoking prevalence among Indigenous Peoples is shaped by a multifaceted interplay of social, cultural, psychological, physiological and historical factors, including socioeconomic disadvantage, early initiation and normalization of tobacco use, and the enduring health impacts of colonization and dispossession (Johnston and Thomas, 2008; Jetty, 2017). In addition, tobacco has ceremonial, medicinal, and spiritual significance within cultural practices for many Indigenous Peoples (Nez Henderson et al., 2022); which complicates cessation efforts and suggests that programs may be more culturally appropriate when they explicitly distinguish between traditional and commercial tobacco (Sanderson et al., 2018).

Over the past two decades, most smoking cessation initiatives designed for Indigenous Peoples have emphasized tailored behavioral and community-based strategies, such as culturally adapted education, individual and group counseling, digital or mobile apps interventions, and incentive-based approaches (Rusk et al., 2023). While these approaches are promising; the effectiveness of tailored interventions in reducing smoking prevalence has been mixed; with studies reporting either significant or non-significant higher rates of smoking cessation; or outcomes comparable to non-tailored supports (Wimbish-Tompkins et al., 2024; Marley et al., 2014; Patten et al., 2023; Whittaker et al., 2011; Carroll et al., 2024).

Pharmacotherapies such as nicotine replacement therapy (NRT), bupropion, varenicline, and cytisine are among the most effective strategies for smoking cessation in the general population (Cahill et al., 2016). However; considerably fewer studies have evaluated the effectiveness of these therapies in Indigenous populations; where cultural; social; and structural contexts may influence both uptake and adherence (Davidson et al., 2010; DiGiacomo et al., 2011). Available evidence suggests that uptake of pharmacotherapies among Indigenous Peoples may be limited due to barriers such as cost, limited access in remote areas, concerns about safety and side effects, trust in health care system, and their perception about the provided medications not aligning with traditional or community-based healing practices (Davidson et al., 2010; Levesque et al., 2024). This review aimed to evaluate randomized clinical trials (RCTs) assessing the effects of pharmacotherapies delivered alone or in combination with behavioral or culturally tailored smoking cessation supports among Indigenous populations.

## 2 Methods

This review was registered in the PROSPERO database (http://www.crd.york.ac.uk/PROSPERO) under record ID number CRD420250591378 on February 24th, 2025. To ensure transparency of reporting, this manuscript adheres to the PRISMA 2020 reporting guidelines (Page et al., 2021).

### 2.1 Eligibility criteria

This review included RCTs of smoking cessation interventions among Indigenous adults from any Indigenous community globally. Trials were eligible if they evaluated pharmacotherapies (alone or combined with behavioral or culturally tailored supports) aimed at reducing or quitting cigarette smoking, compared with placebo, usual care, or alternative interventions. Outcomes of interest were smoking cessation measured by self-report or biochemical verification at any follow-up time point, or reduction in cigarettes smoked per day among participants who continued smoking.

Studies were excluded if they focused exclusively on youth (<18 years), lacked a pharmacotherapy component, did not report smoking cessation outcomes, used non-interventional designs, lacked a comparator, or evaluated only policy, mass media, or social marketing interventions without an individual or community-level cessation component.

### 2.2 Information sources search strategy

A peer reviewed search was conducted on June 26, 2024 in MEDLINE, Embase, CENTRAL, and APA PsycINFO, and September 3, 2024 in Informit Indigenous Collection, Bibliography of Indigenous Peoples in North America, and Global Health. A search update was conducted in all databases except for Informit Indigenous Collection May 28, 2025. This database was excluded from the update because it had been initially searched as part of the trial, was difficult to export from, and the original search did not yield any relevant studies. No limits to language or publication date were applied. The main search concepts comprised of terms related to global Indigenous Peoples and tobacco use cessation and was informed by previously conducted systematic searches and search filters (**see Supplementary file for full search strategies and bibliography**).

### 2.3 Selection process and Data Collection

Search results were exported to Covidence (Melbourne, Australia) and duplicates were eliminated using the platform's duplicate identification feature, supplemented by manual verification. Screening was conducted in two stages: (He et al., 2021) titles and abstracts were independently screened in duplicate (by EB; SS; EL; MS; JM) against eligibility criteria; and (World Health Organization, 2003) full texts of potentially relevant studies were reviewed in duplicate (by EB, SS, EL, MS, JM, SM). Discrepancies were resolved through discussion or adjudication by a third reviewer (EB, SM, JH, HM).

Following study selection, data from eligible RCTs were extracted in duplicate (by EB, SS, MS, JM, SM) using an Excel sheet. Extracted variables included bibliographic details (author, year, title, country), study design, sample size, participant characteristics (Indigenous group, age, sex), intervention type and duration, comparator, and primary outcomes. Information on outcome measurement tools, results (e.g., abstinence or reduction rates), cultural tailoring, reported barriers and facilitators, and funding sources were also collected.

### 2.4 Study Risk of Bias Assessment

Risk of bias for included RCTs was assessed independently by two reviewers (SM, JH) using the Cochrane Risk of Bias 2.0 tool (RoB 2) (JAC et al., 2019) across the five standard domains: bias arising from the randomization process; deviations from intended interventions; missing outcome data; measurement of the outcome; and selection of the reported result. Each study was rated as “low risk of bias;” “some concerns;” or “high risk of bias.” The *robvis* web application (McGuinness and Higgins, 2021) was used to visualize the risk-of-bias assessments.

### 2.5 Synthesis Methods

Due to substantial heterogeneity in study design, intervention delivery, pharmacotherapy type and duration, and outcome measures, a meta-analysis could not be conducted. Findings were synthesized narratively emphasizing smoking-cessation effectiveness and, where reported, treatment adherence and acceptability, adverse events (AEs) and safety.

## 3 Results

### 3.1 Study Selection

**Fig. 1:**
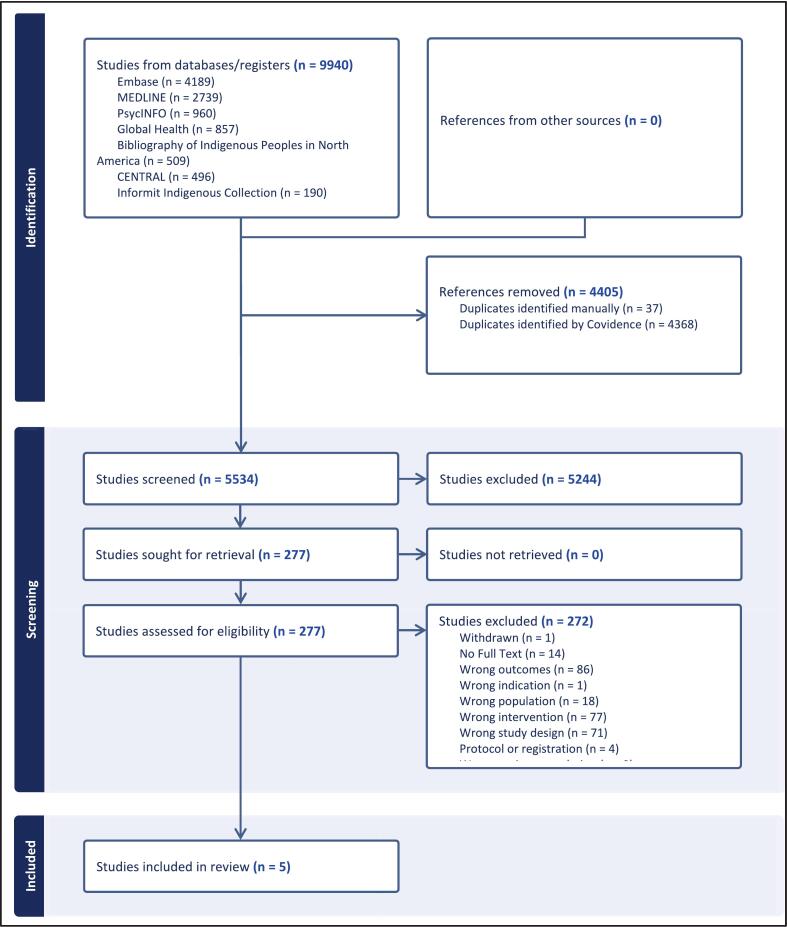
PRISMA flow diagram of study selection for randomized controlled trials of smoking cessation interventions incorporating pharmacotherapy among Indigenous adults identified through database searches conducted in 2024 with an update in May 2025.

The study selection process is presented in a PRISMA flow diagram **(**Fig. 1**)**. The database searches yielded a total of 9940 references. After removal of duplicates, a total of 5534 records were screened, and 277 articles were assessed in full text. Of these, 272 were excluded for reasons detailed in the PRISMA flow diagram, leaving five RCTs that met the inclusion criteria (**Supplemental File, References S1-S5)**.

### 3.2 Main Characteristics of Included Studies

The main characteristics of the included studies are summarized in Table 1. Five randomized controlled trials were published between 2005 and 2021 and were conducted in the United States and New Zealand. Three studies were carried out among AI/AN adults (S1-S3), and two were conducted among Māori populations; one with Māori participants only (S4) and one with Māori and whānau (extended family of Māori) (S5). The total number of participants across studies was 1633, with sample sizes ranging from 103 to 679, and most participants were women (approximately 62% to 76%). Participants were adult daily smokers who were motivated to quit. Pharmacotherapies evaluated included varenicline, cytisine, bupropion, and NRT, administered alone or in combination with culturally tailored behavioral or counseling interventions with most interventions delivered in community or clinical tribal health settings. The duration of pharmacotherapy ranged from 7 to 16 weeks, with follow-up assessments conducted between 3 and 18 months post-quit date. Smoking cessation outcomes were measured as self-reported or biochemically verified 7-day point prevalence abstinence (PPA) or continuous using carbon monoxide or salivary cotinine verification.

**Table 1:** Summary of randomized controlled trials incorporating pharmacotherapy pharmacotherapy for smoking cessation among Indigenous populations in the United States and New Zealand, published between 2005 and 2021, with follow-up ranging from 3 to 18 months.

Author (Year)	Country / Population	Design/ Sample	Intervention / Comparator	Pharmacotherapy	Follow-up	Primary cessation outcome	Other reported outcomes	Funding Source
Choi et al. 2016 (47)	USA, AI/AN adults	RCT (*n* = 463)	ANBL(*n* = 243) + pharmacotherapy OR non-tailored CBP + pharmacotherapy (*n* = 220)	Choice of varenicline, bupropion, or NRT	6 mo	ITT 7-day PPA in ANBL vs CBP *Self-reported:* 12 wks: 27.9% vs 17.4% ⁎, 6-month: 20.1% vs 12.0% ⁎. *Cotinin-verified:* 12 wks: 11.2% vs 8.8%, 6-month: 10.8% vs 6.9%.	60% reduction in cigarettes smoked; mild AEs reported by 15 participants (≈3%) across both groups including sleep disturbance, depression, nausea, constipation, irritability, and skin reactions.	National Institutes of Health (USA)
Smith et al. 2014 (48)	USA, AI/AN adults	RCT (*n* = 103)	CTT (*n* = 50) OR ST (*n* = 53), both with 12 wks Pharmacotherapy	Varenicline	6 mo	ITT 7-day PPA in CTT vs ST *CO-verified:* 3-months: 24.0% vs 24.5%, 6-month: 14.0% vs 22.6%.	High early adherence to varenicline (90% at 1 wk.; 84% at 3 wk.; 32% at 12 wk); three discontinued due to mild AEs (stomach pain, mood changes, nausea); no serious AEs.	University of Wisconsin and Spirit of Eagles Community Network Program
Holt et al. 2005 (50)	New Zealand- Māori	Double-blind RCT (*n* = 134)	pharmacotherapy + culturally adapted counseling (*n* = 88) OR placebo + counseling (*n* = 46)	Bupropion	12 mo	ITT continuous abstinence in treatment vs placebo: *CO-verified:* 3 mo: 44.3% vs 17.4% (RR 2.54, 95% CI 1.30, 5.00); 6 mo: 29.6% vs 10.9% (RR= 2.72, 95% CI 1.12, 6.61); 12 mo: 21.6% vs 10.9%; model-based pooled estimate across all time points RR = 2.44 (95% CI 1.22, 4.88).	Mild AEs; insomnia more common with bupropion (26% vs. 9%); few discontinuations due to minor reactions	GlaxoSmithKline and Novartis grants
Walker et al. 2021 (51)	New Zealand - Māori and whānau	Pragmatic open-label RCT (*n* = 679)	Cytisine (12 wk) + brief support vs. varenicline (12 wk) + brief support	Cytisine vs. Varenicline	6 mo	Continuous abstinence in Cytisine vs. Varenicline group: *Self-reported:* 3 mo (36.7% vs 29.7%); 6 mo (22.9% vs 17.5%); *CO-verified:* 6 mo (12.1% vs 7.9%); 12 mo (16.3% vs 12.4%). 7-day PPA: *Self-reported:* 6 mo (41.4% vs 32.9%) (RR 1.26, 95% CI 1.03, 1.54)	High adherence (96% initiated, 50% completed 12-week course); 44% fewer AEs with cytisine (IRR = 0.56, 95% CI: 0.49, 0.65); fewer withdrawals due to AEs (9% vs 14%); most AEs mild (headache, nausea, sleep disturbance).	Health Research Council of Newzealand
Dignan et al. 2019 (49)	USA, Northern Plains AI	Factorial RCT (*n* = 254)	Combinations of NRT, pre/post counseling, and mHealth (text message–based education) support	NRT vs no NRT (in factorial design)	18 mo	Raw abstinence rates by arm not reported. Logistic regression models were used. At 18 months: NRT associated with increased odds of abstinence (β = −0.79, χ^2^ = 3.77, p = 0.05).	> 90% attrition	National Cancer Institute, USA

⁎ Statistically significant difference between groups (*p* < 0.05). AI/AN, American Indian/Alaska Native; ANBL, All Nations Breath of Life culturally tailored counseling; CBP, current best practices; CCT, Culturally tailored treatment; CO, carbon monoxide; ITT, intention-to-treat; NRT, nicotine replacement therapy; PPA, point-prevalence abstinence; RCT, randomized controlled trial; AEs, adverse events; IRR, incidence rate ratio.

### 3.3 Risk of Bias results

**Fig. 2:**
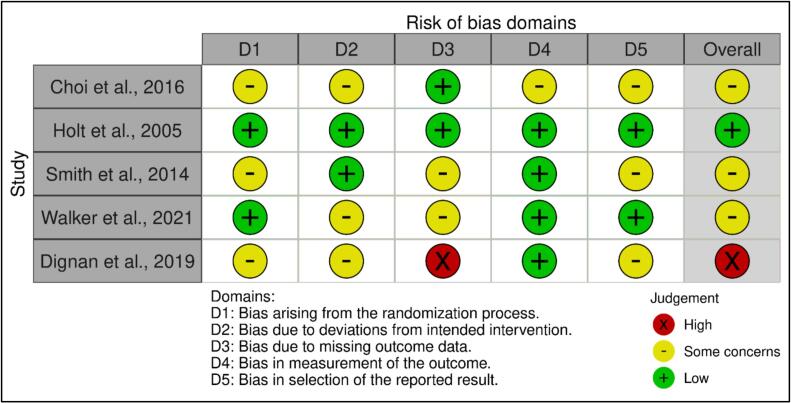
Summary of risk of bias assessments for included randomized controlled trials of smoking cessation interventions incorporating pharmacotherapy among Indigenous adults in the United States and New Zealand (2005–2019), assessed using the Cochrane Risk of Bias 2 (RoB 2) tool.

The risk of bias assessment for the five included RCTs is summarized in Fig. 2. One study was rated at low risk of bias across all domains, and three studies were rated as having some concerns, mainly due to unclear randomization procedures, deviations from intended interventions, incomplete outcome data, and potential selective reporting. One study was rated as having a high risk of bias, primarily due to substantial attrition, with missing data likely related to participants' smoking status. Across studies, measurement of smoking outcomes was generally rated as low to some concerns, as abstinence was verified using biochemical measures (carbon monoxide or cotinine) and standardized thresholds.

### 3.4 Description of interventions with pharmacotherapy component

Across the included trials, pharmacotherapies were delivered either universally, compared with placebo, or examined within factorial designs, and were commonly paired with behavioral or culturally tailored supports. Two studies provided pharmacotherapies to all participants in combination with culturally tailored or non-tailored behavioral interventions. In a trial by Choi et al. (S1) among AI adults in the United States, participants were offered a choice of pharmacotherapy, including varenicline, bupropion, or NRT (gum, patch, or lozenge), and were randomized to either the culturally tailored All Nations Breath of Life (ANBL) program or a non-tailored current best practices program over 6 months. The ANBL intervention incorporated group and individual counseling delivered by an AI facilitator, motivational interviewing, educational materials reflecting Indigenous perspectives, and tailored participant incentives, whereas the comparison group received standard, non-tailored cessation support. In the START trial, Smith et al. (S2) randomized AI/AN adults to receive either a culturally tailored or standard counseling intervention, with both groups receiving varenicline pharmacotherapy for 12 weeks. The culturally tailored arm incorporated community-developed elements, including discussions distinguishing traditional and commercial tobacco use, culturally specific educational resources, and Indigenous metaphors to support cessation.

Three additional studies evaluated pharmacotherapies more directly. A pragmatic open-label randomized controlled trial in New Zealand compared cytisine (1.5 mg every 2–6 h for 25 days, then maintenance to week 12) with varenicline (0.5–1 mg once or twice daily, titrated over 12 weeks) among Māori and whānau participants, with both groups receiving brief cessation advice by the physician and referral to community-based cessation services (S5). A double-blind, placebo-controlled trial among Māori adults evaluated bupropion (150 mg once daily for 3 days, then 150 mg twice daily for 7 weeks) combined with culturally adapted counseling, compared with placebo plus equivalent counseling (S4). Finally, a factorial randomized trial among Northern Plains AIs examined smoking cessation strategies combining NRT, pre- and post-cessation counseling education, and mobile health support delivered via text messages. Participants were assigned to multiple study arms representing different combinations and intensities of these components, allowing assessment of both individual and combined effects within a culturally embedded intervention framework (S3).

### 3.5 Smoking Cessation Outcomes

Smoking cessation outcomes varied across trials, reflecting differences in pharmacotherapy type, comparator conditions, outcome definitions, and follow-up duration. In the Choi et al. (S1) study, the intention-to-treat (ITT) self-reported 7-day PPA was significantly higher in the ANBL group compared to the CBP control at both 12 weeks (27.9% vs. 17.4%; OR = 1.87, 95% CI: 1.07, 3.26) and 6 months (20.1% vs. 12.0%; OR = 1.91, 95% CI: 1.07, 3.42), although cotinine-verified abstinence rates did not differ significantly between groups at 12 weeks (11.2% vs. 8.8%) or 6 months (10.8% vs. 6.9%), likely due to limited statistical power resulting from low sample sizes for biochemical verification. For study responders only, self-reported 7-day PPA was higher in the ANBL group compared to the CBP control at 12 weeks (49.0% vs. 30.9%; OR = 2.16, 95% CI: 1.16, 4.04) and at 6 months (35.9% vs. 28.3%; OR = 1.67, 95% CI: 0.82, 3.41), although the difference at 6 months was not statistically significant. At the 12-week follow-up, among participants who had not quit smoking, cigarette consumption decreased by approximately 60% from baseline across participants in both study groups. In the Smith et al. (S2) study, there were no statistically significant differences in biochemically-confirmed 7-day PPA between the culturally tailored (CTT) and standard (ST) groups at either 3 or 6 months, for both ITT (24.5% vs. 24.0% and 14.0% vs. 22.6%) and responder-only analyses (57.1% vs. 40.6% and 42.9% vs. 41.2%), respectively. Across both groups that used pharmacotherapy, the overall ITT abstinence rate was 24% at 3 months and 20% at 6 months, while responder-only rates were 47% and 42%, respectively, indicating the influence of participant retention and continued engagement on observed quit outcomes.

In the study by Walker et.al (S5), self-reported abstinence outcomes were generally higher in the cytisine group, though most differences did not reach statistical significance. At 3 months and 6 months, continuous abstinence for cytisine vs varenicline was 36.7% vs 29.7% (RR = 1.23, 95% CI: 0.99, 1.53), and 22.9% vs 17.5% (RR = 1.31, 95% CI: 0.97, 1.77), respectively. Additionally, self-reported 7-day PPA at 6 months was also significantly higher in the cytisine group (41.4% vs 32.9%; RR = 1.26, 95% CI: 1.03, 1.54). However, the carbon monoxide (CO)-verified 6-month continuous abstinence was only 12.1% in the cytisine group compared to 7.9% in the varenicline group (RR = 1.55, 95% CI: 0.97, 2.46), suggesting possible overestimation of abstinence in self-reported outcomes. Overall, the trial demonstrated that cytisine was at least as effective as varenicline in supporting smoking cessation in this population. In the study by Holt et al. (S4), at 3 months, participants receiving bupropion achieved a significantly greater rate of CO-verified continuous abstinence compared to those receiving placebo group (44.3% vs 17.4%, RR = 2.54, 95% CI: 1.30, 5.00). Although the difference at 12 months did not reach statistical significance (21.6% vs. 10.9%, RR = 1.99, 95% CI: 0.79, 5.00), a repeated measures model with a change point at 26 weeks showed a sustained advantage for bupropion (RR = 2.44, 95% CI: 1.22, 4.88). These findings suggest that bupropion, when paired with culturally tailored counseling, may offer benefits for long-term smoking abstinence in this population. In the Dignan et al. (S3) trial, unlike the other pharmacotherapy trials, raw abstinence rates by intervention group were not reported. Instead, the investigators presented logistic regression models, which showed that use of NRT increased the odds of not smoking at the 18-month follow-up (β = −0.79, χ^2^ = 3.77, *p* = 0.05). While these findings suggest potential benefits of NRT in this population, interpretation is limited by the extremely high attrition, with only 16 of 254 participants remaining enrolled at 18 months (> 90% attrition), which greatly reduces confidence in the result.

Overall, the included trials demonstrated modest cessation outcomes, with consensus showing improvements associated with pharmacotherapy use or combined culturally tailored approaches.

### 3.6 Adherence to Pharmacotherapy and AEs

Adherence and AEs data were reported variably across the included trials. In the Walker et al. (S5) study comparing cytisine and varenicline, 96% of participants collected all or at least part of their prescription and 50% collected their medication for the full 12-week course of the intervention. Cytisine was associated with 44% fewer self-reported AEs (incidence rate ratio = 0.56, 95% CI: 0.49, 0.65) and fewer withdrawals due to AEs (9% vs. 14% in the varenicline group), suggesting better tolerability. The authors also noted that some participants expressed a preference for cytisine because it was plant-based and perceived as more ‘natural,’ which may have contributed to greater acceptability and retention in this group. A smaller proportion of AEs were considered treatment-related in the cytisine group compared to varenicline (41% vs. 57%), and serious AEs were uncommon and not judged to be related to study medications. The most frequently reported AEs in both groups were mild and consistent with the known safety profiles of varenicline and cytisine (Cahill et al., 2016). In the Holt et al. (S4) trial, most side effects were mild and self-limiting. Participants receiving bupropion were more likely to experience insomnia compared with those in the placebo group (26% vs. 9%; risk ratio = 3.0, 95% CI: 1.1, 8.2). Three participants discontinued treatment due to rash, but no serious AEs were reported.

In the Smith et al. (S2) trial, adherence to varenicline was initially high but rates were not maintained throughout the 12-week course, with 90.2% of participants taking the medication at one week post-quit, 84.0% at 3 weeks, and 32.1% at 12 weeks, with no difference between the culturally tailored and standard treatment groups. No serious AEs were reported. Three participants discontinued varenicline early due to side effects, including stomach pain, mood changes, and nausea, although all remained in the study. In the Choi et al. (S1) study, adherence to pharmacotherapy was not reported, but overall 83% of participants opted for a pharmacologic aid, including 40% using NRT, 35% varenicline, 5% bupropion, and 3% a combination of bupropion and NRT, while 17% chose no pharmacotherapy. The AEs were minimal, reported by 15 participants across both study arms (7 in the ANBL and 8 in the CBP group). In the Dignan et al. (S3) trial, no quantitative data on adherence to pharmacotherapy or AEs were reported. However, the authors noted that use of NRT was associated with greater abstinence among participants who continued in the study through the 18-month follow-up, suggesting a potential benefit of pharmacologic support among those who remained engaged in the intervention.

Overall, where reported, early adherence was high across trials and AEs were generally mild and consistent with known effects of cessation medications (Ebbert et al., 2021), with no serious safety concerns reported.

## 4 Discussion

This systematic review synthesized evidence from five RCTs using pharmacotherapies for smoking cessation among Indigenous populations in the United States and New Zealand. The interventions examined NRT, varenicline, bupropion, and cytisine, delivered either alone or in combination with behavioral or culturally tailored supports. Across the included studies, pharmacotherapy use was associated with self-reported 7-day PPA ranging from 12.0% to 41.4%, biochemically verified 7-day PPA ranging from 6.9% to 22.6%, and biochemically verified continuous abstinence ranging from 7.9% to 29.6% at 6 months. The available evidence remains limited and heterogeneous, reflecting variability in trial design, comparators, and outcome measurement.

Two trials (S1, S2) provided pharmacotherapy in both study arms, such that comparisons primarily reflected differences in behavioral components rather than medication effects alone. One trial demonstrated modest advantages for the culturally tailored arm based on self-report, whereas the other found no differences between conditions. These findings highlight the potential role of cultural and behavioral support, but they do not provide evidence on the independent effectiveness of pharmacotherapy. Cytisine was non-inferior to varenicline, but the absence of a placebo group limited assessment of absolute efficacy (S5), while evidence for NRT was constrained by substantial (over 90%) attrition (S3). Direct evidence of pharmacotherapy efficacy was strongest in the placebo-controlled trial of bupropion (S4), which demonstrated significant short-term benefits. Although the advantage was not statistically significant at 12 months, participants receiving bupropion were still nearly twice as likely to remain abstinent compared with those receiving placebo. These findings provide only limited but suggestive evidence of pharmacotherapy effectiveness in Indigenous individuals, with outcomes appearing to depend heavily on adherence to the intervention, participant retention, and cultural acceptability.

Use of pharmacotherapy has been widely recommended in international clinical guidelines as an effective aid to smoking cessation, particularly when combined with behavioral support (Ministry of Health, 2021; Mir et al., 2025). Extensive evidence from general population studies consistently demonstrates that pharmacotherapies substantially improve smoking cessation outcomes. Systematic reviews indicate that NRT increases quit rates by approximately 50–60% compared with placebo (Hartmann-Boyce et al., 2018), bupropion increases quit success by about 62% (Hughes et al., 2014); varenicline more than doubles quit success; and cytisine may increase quit rates up to fourfold; although based on more limited evidence (Cahill et al., 2016). In contrast to the extensive and consistent evidence in the general population, the evidence among Indigenous Peoples remains sparse and heterogeneous. While the available RCTs indicate that pharmacotherapies can support cessation, their effects were often difficult to disentangle from concurrent tailored cultural and behavioral supports, and sustained abstinence was challenging to achieve. This reflects both methodological limitations and the broader social and cultural determinants of health that impact smoking behaviors in Indigenous communities.

Cross-national evidence indicates variability in the use of smoking cessation pharmacotherapies among Indigenous populations, with cost and access consistently identified as major barriers (DiGiacomo et al., 2011; Johnston and Thomas, 2010; Boles et al., 2009). In this review, provision of pharmacotherapies at no cost was associated with high uptake across trials (S1, S2, and S5). Similar uptake patterns have been reported in Indigenous cessation programs outside randomized trials, including a tailored tobacco treatment program within AI/AN health services in Minnesota (81% pharmacotherapy use) and a mail-out NRT intervention among Aboriginal and Torres Strait Islander people (>90% uptake and more than half maintained consistent use throughout the intervention period) (D'Silva et al., 2011, Kennedy et al., 2025). Collectively, these findings underscore the importance of reducing cost-related barriers to pharmacotherapy access; however, the modest cessation outcomes observed despite free provision highlight the multifactorial nature of smoking behaviors in Indigenous populations.

Beyond structural barriers, adherence to treatment and sustained engagement also limited effectiveness across trials. In Smith et al. (S2), disengagement from treatment was higher than expected, and household smoking emerged as an important barrier, with nearly 70% of participants with a smoking spouse or partner and more than 70% with immediate or extended family members who smoked being less likely to achieve cessation at six months. In Choi et al. (S1), retention at six months was higher in the culturally tailored ANBL arm compared with the non-tailored control, suggesting that greater group cohesion, social support, and cultural relevance may enhance engagement. In contrast, high attrition was also observed in other trials, including Holt et al. (S4), where 36% of participants in the bupropion group and 52% in the placebo group were lost to follow-up, and Walker et al. (S5), where only 39% of participants remained engaged at six months despite high initial uptake. Collectively, these findings indicate that while pharmacotherapies are feasible and acceptable, high attrition remains a consistent limitation, underscoring the importance of strategies to strengthen adherence and long-term retention.

This review is limited by the small number of available RCTs using pharmacotherapies for smoking cessation, most of which were conducted in the United States and New Zealand, restricting the generalizability of findings to Indigenous Peoples in other regions. Considerable heterogeneity in interventions, comparators, and outcome measures further constrained synthesis. Quantitative synthesis was not performed due to limited overlapping data. Given this limitation, we drew on qualitative contextual information across trials including intervention uptake, adherence, cultural relevance, and retention challenges to support interpretation of the findings. High rates of attrition across the trials also reduce confidence in the findings, as participants lost to follow-up were generally assumed to be smoking, a practice that may underestimate true quit rates. Despite high quality and certainty evidence for pharmacotherapy in the general population, effectiveness among Indigenous Peoples appear modest and variable. Beyond the limited number of trials, this may also reflect social, cultural, and structural contexts that influence uptake, adherence, and sustained abstinence. To advance the field, future randomized trials should address several recurring gaps identified in this review. Priority areas include designs that better isolate the independent and additive effects of different pharmacotherapies, alone and in combination with behavioral or culturally tailored supports; greater consistency in outcome definitions and follow-up time points; and strategies to improve retention that are co-developed with communities, such as family- or household-based approaches, flexible delivery models, and sustained trust-based engagement. Importantly, trials should be community-led and culturally grounded, ensure free and reliable access to medications, and be conducted across more diverse Indigenous contexts globally.

## 5 Conclusion

Overall, these findings indicate that pharmacotherapies were generally acceptable and well tolerated among Indigenous participants, with AEs reported as mild and consistent with known safety profiles. The limited number of available RCTs provides suggestive evidence that these treatments can support smoking cessation in Indigenous populations. Interpretation is constrained by substantial heterogeneity, as different pharmacotherapies were used across studies and often delivered alongside behavioral or culturally tailored components, making it difficult to isolate their independent effects. The small number of studies also reflects ongoing inequities in cessation research, with Indigenous Peoples remaining underrepresented despite disproportionately high smoking prevalence. Future research should prioritize community-led trials that combine pharmacotherapies with culturally tailored support and ensure free, consistent access to medications.

## CRediT authorship contribution statement

**Salma Mahmoodianfard:** Writing – review & editing, Writing – original draft, Methodology, Formal analysis, Data curation, Conceptualization. **Emma Lynn Bates:** Writing – review & editing, Writing – original draft, Methodology, Data curation, Conceptualization. **Javad Heshmati:** Writing – review & editing, Methodology, Conceptualization, Data curation, Writing – original draft. **Maalik Sheraly:** Writing – review & editing, Data curation, Formal analysis, Investigation, Methodology. **Jake Miles:** Writing – review & editing, Data curation, Formal analysis, Investigation, Methodology. **Sarah Visintini:** Writing – review & editing, Resources, Investigation, Methodology, Writing – original draft, Validation. **Richard San Cartier:** Writing – review & editing, Investigation, Formal analysis, Methodology, Validation. **Hassan Mir:** Writing – review & editing, Writing – original draft, Supervision, Methodology, Investigation, Funding acquisition, Conceptualization, Data curation, Formal analysis.

## Declaration of competing interest

The authors declare the following financial interests/personal relationships which may be considered as potential competing interests: Hassan Mir reports financial support was provided by Canadian Cancer Society. Salma Mahmoodianfard reports financial support was provided by Canadian Institutes of Health Research. If there are other authors, they declare that they have no known competing financial interests or personal relationships that could have appeared to influence the work reported in this paper.

## Data Availability

Data will be made available on request.
